# Phosphorylated STAT3 as a potential diagnostic and predictive biomarker in ALK^-^ ALCL vs. CD30^high^ PTCL, NOS

**DOI:** 10.3389/fimmu.2023.1132834

**Published:** 2023-06-14

**Authors:** Chenxi Xiang, Wanna Wu, Meiting Fan, Zhen Wang, Xiaoli Feng, Cuiling Liu, Jia Liu, Guangzhen Liu, Lei Xia, Haipeng Si, Ying Gu, Nian Liu, Dan Luo, Yubo Wang, Dongshen Ma, Shimin Hu, Hui Liu

**Affiliations:** ^1^ Department of Pathology, The Affiliated Hospital of Xuzhou Medical University, Xuzhou, China; ^2^ Department of Pathology, Xuzhou Medical University, Xuzhou, China; ^3^ Department of Pathology, The First Affiliated Hospital and School of Clinical Medicine of Guangdong Pharmaceutical University, Guangzhou, China; ^4^ Department of Pathology, The First Affiliated Hospital of Nanjing Medical University, Nanjing, China; ^5^ Department of Pathology, National Cancer Center and National Clinical Research Center For Cancer and Cancer Hospital, Chinese Academy of Medical Sciences and Peking Union Medical College, Beijing, China; ^6^ Department of Pathology, School of Basic Medical Sciences and Third Hospital, Pekin University Health Science Center, Beijing, China; ^7^ Department of Pathology, The Affiliated Hospital of Nanjing University of Chinese Medicine, Nanjing, China; ^8^ Department of Hematopathology, The University of Texas MD Anderson Cancer Center, Houston, TX, United States

**Keywords:** anaplastic large cell lymphoma, periphearal T cell lymphoma, not otherwise specified, CD30, pSTAT3

## Abstract

**Aims:**

The differential diagnosis between ALK-negative anaplastic large cell lymphoma (ALK^-^ ALCL) and peripheral T-cell lymphoma, not otherwise specified (PTCL, NOS) with high expression of CD30 (CD30^high^) are essential. However, no reliable biomarker is available in daily practice except CD30. STAT3 is characteristically activated in ALCL. We aimed to investigate whether the status of STAT3 phosphorylation could help the differential diagnosis.

**Methods:**

The status of phosphorylation of STAT3 was examined using two antibodies against pSTAT3-Y705 and pSTAT3-S727 by immunohistochemistry in ALK^+^ ALCL (n=33), ALK^-^ ALCL (n=22) and PTCL, NOS (n=34). Ten PTCL, NOS with diffuse CD30 expression were defined as CD30^high^ PTCL, NOS. Flowcytometric analysis were performed to evaluate the expression of pSTAT3-Y705/S727 in PTCL, NOS (n=3).

**Results:**

The median H-scores of pSTAT3-Y705 and S727 were 280 and 260 in ALK^+^ ALCL, 250 and 240 in ALK^-^ ALCL, and 45 and 75 in CD30^high^ subgroup, respectively. Using H score of 145 as the cutoff value, pSTAT3-S727 alone distinguished between ALK^-^ ALCL and CD30^high^ PTCL, NOS with a sensitivity of 100% and specificity of 83%. Additionally, pSTAT3-S727, but not pSTAT3-Y705, was also expressed by background tumor-infiltrating lymphocytes (S727_TILs_) in PTCL, NOS. PTCL, NOS patients with high S727_TILs_ H score had a favorable prognosis than those with no TILs (3-year OS rate: 43% vs. 0, *p*=0.013) or low S727_TILs_ (3-year OS rate: 43% vs. 0, *p*=0.099). Flowcytometric analysis revealed that of the three patients investigated, two had enhanced pSTAT-S727 signals in neoplastic cell populations, and all three patients were negative for pSTAT3-Y705 expression in both tumor cells and background lymphocytes.

**Conclusions:**

pSTAT3-Y705/S727 can be used to help distinguish ALK^-^ ALCL from CD30^high^ PTCL, NOS and pSTAT3-S727 expression by TILs predicts the prognosis of a subset of PTCL, NOS.

## Introduction

Anaplastic large cell lymphoma (ALCL), is a type of lymphoma that can be classified into two subtypes: ALK^+^ and ALK^-^. Both subtypes are characterized by the presence of large anaplastic cells with diffuse and strong expression of CD30 on the cell membrane and in the Golgi region (1, 2). The diagnosis of ALK^+^ ALCL is usually straightforward. However, for most cases of ALK^-^ ALCL, no unifying genetic abnormality has been identified, making the diagnosis more reliant on morphology features and CD30 expression (3).

Despite its strong expression on anaplastic cells in ALK^+^ and ALK^-^ ALCL, CD30 is not a specific marker for these malignancies. In fact, a variety of T- or B-cell lymphomas, as well as reactive lymphoproliferative disorders, and non-hematopoietic lesions, may also express CD30 to varying degrees. Among these entities, peripheral T-cell lymphoma, not otherwise specified (PTCL, NOS) with CD30 expression can be particularly difficulty to distinguish from ALK^-^ ALCL. Although most cases of PTCL, NOS are negative or only weakly positive for CD30 in only a minority of tumor cells (4–6), a small subset exhibit diffuse and strong CD30 expression(CD30^high^ PTCL, NOS). In such cases, the distinction between ALK^-^ ALCL and PTCL, NOS becomes ambiguous, and the diagnosis may be subjective (5, 7).

Differentiating between these two conditions is clinically important since patients with ALK^-^ ALCL typically have better overall survival and respond more favorably to Brentuximab vedotin compared to those with PTCL, NOS (8). Unfortunately, due to the lack of widely accepted diagnostic criteria or reliable biomarkers to differentiate between ALK^-^ ALCL and CD30^high^ PTCL, NOS, accurately diagnosing these two conditions remains a challenge and requires careful consideration of clinical, morphological, and immunophenotypic features by an experienced pathologist.

Aberrant activation of the signal transducer and activator of transcription 3 (STAT3) has been identified as a key oncogenic event in both ALK*^+^* and ALK*^-^* ALCL (9–15). In ALK^+^ ALCL, the NPM::ALK fusion protein drives STAT3 activation by promoting the phosphorylation of STAT3 (15). In ALK^-^ ALCL, the STAT3 pathway can be activated by alternative upstream signals, such as *JAK1/STAT3* mutations (5, 16), NFkB2::ROS1 and NFkB::TYK2 fusions (9). However, *JAK1/STAT3* mutations are not commonly observed in PTCL, NOS (9). These findings raised the question of whether the activation status of STAT3 could be used to distinguish ALK^-^ ALCL from CD30^high^ PTCL, NOS. Notably, phosphorylation of STAT3 at tyrosine 705 (Y705) was found to be positive in 84-93% of ALK^+^ ALCL and 47-57% of ALK^-^ ALCL (17, 18). The phosphorylation status of STAT3 in PTCL, NOS remains largely unknown, and the phosphorylation of STAT3 at another activation site, serine 727 (S727) has not been well studied, in contrast to the frequent observation of positive phosphorylation at Y705 in both ALK*^+^* and ALK*^-^* ALCL.

This study aimed to develop an immunohistochemistry (IHC)-based classifier using two commercially available antibodies targeting different phosphorylation sites of STAT3, namely Y705 and S727. The goal was to determine whether the phosphorylation status of these residues could serve as a biomarker for distinguishing between ALK^-^ ALCL and CD30^high^ PTCL, NOS. Furthermore, the study investigated whether the presence of phosphorylated STAT3 (pSTAT3) could predict clinical outcomes in patients with ALCL or PTCL, NOS.

## Materials and methods

### Patient selection

CD30 expression was retrospectively investigated in cases diagnosed with ALK^+^ ALCL, ALK^-^ ALCL, and PTCL, NOS from five different institutions. Two hematologists independently reviewed the diagnosis of each case based on 2017 WHO classification criteria and finally included 89 patients from four different medical centers (The Affiliated Hospital of Xuzhou Medical University, The First Affiliated Hospital of Nanjing Medical University, Chinese Academy of Medical Sciences Cancer Hospital, Third Hospital of Pekin University Health Science Center), comprising 33 cases of ALK^+^ ALCL, 22 cases of ALK^-^ ALCL, and 34 cases of PTCL, NOS (4). Clinicopathologic data and follow-up information were collected for each case.

### Immunohistochemistry and pSTAT3-Y705 and pSTAT3-S727 semi-quantification

The antibodies used to detect pSTAT3-Y705 (ab171358) and pSTAT3-S727 (ab32143) were from Abcam (Cambridge, UK). Positive IHC staining for pSTAT3-Y705 and pSTAT3-S727 was defined as clear nuclear staining with a distinct background. The secondary antibody was Polymer HRP-Goat anti-Rabbit/Mouse Secondary Antibody (PV-8000) from ZSGB-BIO (Beijing, China). The extent of staining was categorized into three grades based on the percentage of positive cells (<30%, 30-70%, and >70%). The intensity of staining was evaluated as weak (1+), moderate (2+), or strong (3+). Semi-quantitative evaluation of IHC was performed using the H-score, which is calculated as 300 × (% cells 3+) + 200 × (% cells 2+) + 100 × (% cells 1+). Tumor-infiltrating lymphocytes (TILs) was defined as lymphocytes interspersing within the tumor area. The expression of pSTAT3-S727 in TILs (S727_TILs_) was evaluated by multiplying the H-score of S727 in TILs by the percent of TILs in the tumor area: H-score × TILs % × 100.

### Flowcytometric assay

Flowcytometric assay was performed on three PTCL, NOS cases, using one excised tonsil case as control. All PTCL, NOS cases were diagnosed in our laboratory using a T-cell lymphoma panel recommended by Euroflow and excluded from PTCL of T follicular helper (Tfh) cell phenotype (19) on a Cytek Spectrum Flowcytometer (NL-CLC, V16-B14). The detection of pSTAT3-Y705 was by using commercially available antibody (Catalog No: 612569) from BD Pharmingen (BD Bioscience, USA) and pSTAT3-S727 by conjugating fluorescence tag (FlexAble Coralite V405 Antibod Labeling Kit For Rabbit IgG, Cat No: KFA006) with pSTAT3-S727 (ab32143, Abcam) from Abcam (Cambridge, UK), in an 8-color panel consisting of pSTAT3-Y705-FITC, pSTAT3-S727-Corolite V405, CD3-ECD (Catlog No: A07748, Beckman Coulter), CD8-PE-Cy7 (Catlog No: 6607102, Beckman Coulter), CD10-PE-Cy5 (Catlog No: A07761, Beckman Coulter), CD19-PE-Cy5.5 (Catlog No: A66328, Beckman Coulter), CD45-cFlourV547 (Catlog No: R7-10011, Cytek), CD4-cFlourV610 (Catlog No: R7-20073, Cytek) and PD-1-BV750 (Catlog No: 329966, Biolegend).

### DNA sequencing

The DNA sequencing was performed as described (20). Specifically, probes targeting at 103 genes (**Table S2**) related with T/NK cell lymphoma were used to capture the target genes for DNA sequencing. GENESEEQ Technology Inc. (Nanjing, China) performed the bioinformatics analysis.

### Statistical analysis

All tests were two-sided, and *p* values <0.05 were considered significant. Receiver operating characteristic (ROC) curves were plotted by using the “roc” function in the pROC package (RMS R package). The optimal cutoff of pSTAT3-Y705 and -S727 was derived by calculating the Youden index (sensitivity + specificity - 1). The significance of the associations of pSTAT3-Y705 and -S727 with other categorical variables were evaluated using Fisher’s exact test, Mann-Whitney U test, or the Kruskal-Wallis test. The correlation between two continuous quantitative variables was assessed using Spearman’s rank correlation coefficient. Kaplan-Meier curves were plotted using the “survival” and “survminer” packages of the RMS R package to compare survival data, and significance was assessed using the log-rank test.

## Results

### Baseline clinical characteristics of the study cohort

The study cohort comprised 89 patients, with 33 diagnosed with ALK^+^ ALCL, 22 with ALK^-^ ALCL, and 34 with PTCL, NOS (**Table 1**). Of the PTCL, NOS cases, 10 with CD30 expression in 80% tumor cells were classified as CD30^high^ PTCL, NOS (10/34, 29%). The remaining PTCL, NOS cases (24/34, 71%) were classified as CD30^-/low^ PTCL, NOS. The clinicopathological features of CD30^high^ PTCL, NOS patients were similar to those of ALK^-^ ALCL patients, although CD30^high^ PTCL, NOS patients tended to have lower platelet counts (*p*=0.0913) and to have a lower rate of complete or partial remission (CR/PR) (*p*=0.0873) (**Table 2**). Within the PTCL, NOS group, there was no significant difference in these clinicopathological parameters between CD30^high^ and CD30^-/low^ subgroups (**Table 2**). Additionally, PTCL, NOS (n=5) and ALK^-^ ALCL (n=1) cases which were challenging to make a definite diagnosis based on only morphologic features and immunophenotypes were subjected to DNA sequencing using a 103-gene panel that covers genes associated with the oncogenesis and treatment of T/NK lymphoma, and the results were provided in **Table S1**. The whole exosome of JAK1 and STAT3 were investigated and none of six patients tested positive for JAK1 and STAT3 mutations. Of the five PTCL, NOS patients, three had high expression of pSTAT3-Y705 and four had high expression of pSTAT3-S727, and none of them tested positive for JAK1/STAT3 mutations (**Table S1**).

**Table 1:** Summary of clinicopathological information of the included patients.

	ALK^+^ ALCL (n=33)	ALK^-^ ALCL (n=22)	PTCL, NOS (n=34)	*p* value^a^	*p* value^b^	*p* value^c^
Male	27/33 (82%)	13/21 (62%)	22/34 (65%)	0.123	0.168	>0.9999
Age≥60	3/33 (9%)	12/21 (57%)	20/34 (59%)	0.0003	<0.0001	>0.9999
Median age (range)	26 (5–70)	63 (29-77)	64.5 (28-85)	< 0.001^#^	<0.001^#^	0.349^#^
ECOG PS>1	5/31 (16%)	3/17 (18%)	7/33 (21%)	>0.9999	0.752	> 0.9999
B symptoms	14/31 (45%)	7/21 (33%)	18/33 (55%)	0.565	0.617	0.166
Extranodal lesions	11/33 (33%)	13/21 (62%)	14/34 (41%)	0.052	0.615	0.171
Platelet count (<150×10^9^/L)	1/32 (3%)	3/18 (17%)	9/33 (27%)	0.127	0.013	0.502
Hemoglobin<120g/L	13/32 (41%)	7/18 (39%)	18/33 (55%)	>0.9999	0.324	0.382
Albumin<35g/L	10/19 (53%)	5/10 (50%)	20/27 (74%)	>0.9999	0.209	0.240
High serum beta-2 microglobulin level*	7/16 (44%)	1/7 (14%)	12/16 (75%)	0.345	0.149	0.019
LDH>250U/L	14/27 (52%)	5/15 (33%)	19/32 (59%)	0.337	0.607	0.125
EBV DNA≥10^4^ copies/mL	0/15 (0%)	0/10 (0%)	5/19 (26%)	>0.9999	0.053	0.134
Bone marrow involvement	0/16 (0%)	2/8 (25%)	5/17 (29%)	0.104	0.045	0.234
Spleen and liver involvement	1/18 (6%)	1/8 (13%)	7/21 (33%)	0.529	0.049	0.381
Advanced stage (III/IV)	15/30 (50%)	10/17 (59%)	26/33 (79%)	0.762	0.02	0.187
High IPI risk (3-5)	4/31 (13%)	3/17 (18%)	15/33 (45%)	0.686	0.006	0.067
Therapy response (CR/PR)	20/28 (71%)	8/16 (50%)	1/32 (3%)	0.2	< 0.0001	0.0003

CR, complete remission; PR, partial remission;

^a^ALK^+^ ALCL vs. ALK^-^ ALCL;

^b^ALK^+^ ALCL vs. PTCL, NOS;

^c^ALK^-^ ALCL vs. PTCL, NOS;

^#^Mann-Whitney U P value;

^*^>2400ng/mL.

**Table 2:** Summary of clinicopathological information of ALCL^-^ ALCL and CD30^-/low^ PTCL, NOS patient.

	ALK^-^ ALCL (n=22)	CD30^high^ PTCL, NOS (n=10)	CD30^-/low^ PTCL, NOS (n=24)	*p* value^a^	*p* value^b^
Male	13/21 (62%)	5/10 (50%)	17/24 (71%)	0.701	0.271
Age≥60	12/21 (57%)	5/10 (50%)	15/24 (63%)	>0.9999	0.704
Median age (range)	63 (29-77)	65 (32-85)	59 (28-76)	0.950^#^	0.423^#^
ECOG PS>1	3/17 (18%)	3/10 (30%)	4/23 (17%)	0.638	0.642
B symptoms	7/21 (33%)	4/10 (40%)	14/23 (61%)	>0.9999	0.448
Extranodal lesions	13/21 (62%)	4/10 (40%)	10/24 (42%)	0.441	>0.9999
Platelet count (<150×10^9^/L)	3/18 (17%)	5/10 (50%)	4/23 (17%)	0.091	0.09
Hemoglobin<120g/L	7/18 (39%)	6/10 (60%)	12/23 (52%)	0.433	>0.9999
Albumin<35g/L	5/10 (50%)	2/4 (50%)	18/23 (78%)	>0.9999	0.269
High serum beta-2 microglobulin level^*^	1/7 (14%)	1/3 (33%)	11/13 (85%)	>0.9999	0.136
LDH>250U/L	5/15 (33%)	5/10 (50%)	14/22 (64%)	0.442	0.6993
EBV DNA≥10^4^ copies/mL	0/10 (0%)	1/6 (17%)	4/13 (31%)	>0.375	>0.9999
Bone marrow involvement	2/8 (25%)	1/3 (33%)	4/14 (29%)	>0.9999	>0.9999
Spleen and liver involvement	1/8 (13%)	1/2 (50%)	6/19 (32%)	>0.378	>0.9999
Advanced stage (III/IV)	10/17 (59%)	8/10 (80%)	18/23 (78%)	0.406	>0.9999
High IPI risk (3-5)	3/17 (18%)	3/10 (30%)	12/23 (52%)	0.638	0.283
Therapy response (CR/PR)	8/16 (50%)	1/10 (10%)	0/22 (0%)	0.087	0.313

CR, complete remission; PR, partial remission;

^a^ALK^-^ ALCL vs. CD30^high^ PTCL, NOS;

^b^CD30^high^ vs. CD30^-/low^ PTCL, NOS;

^#^Mann-Whitney U P value;

^*^>2400ng/mL.

### pSTAT3-Y705 and -S727 as a diagnostic biomarker

**Figure 1:**
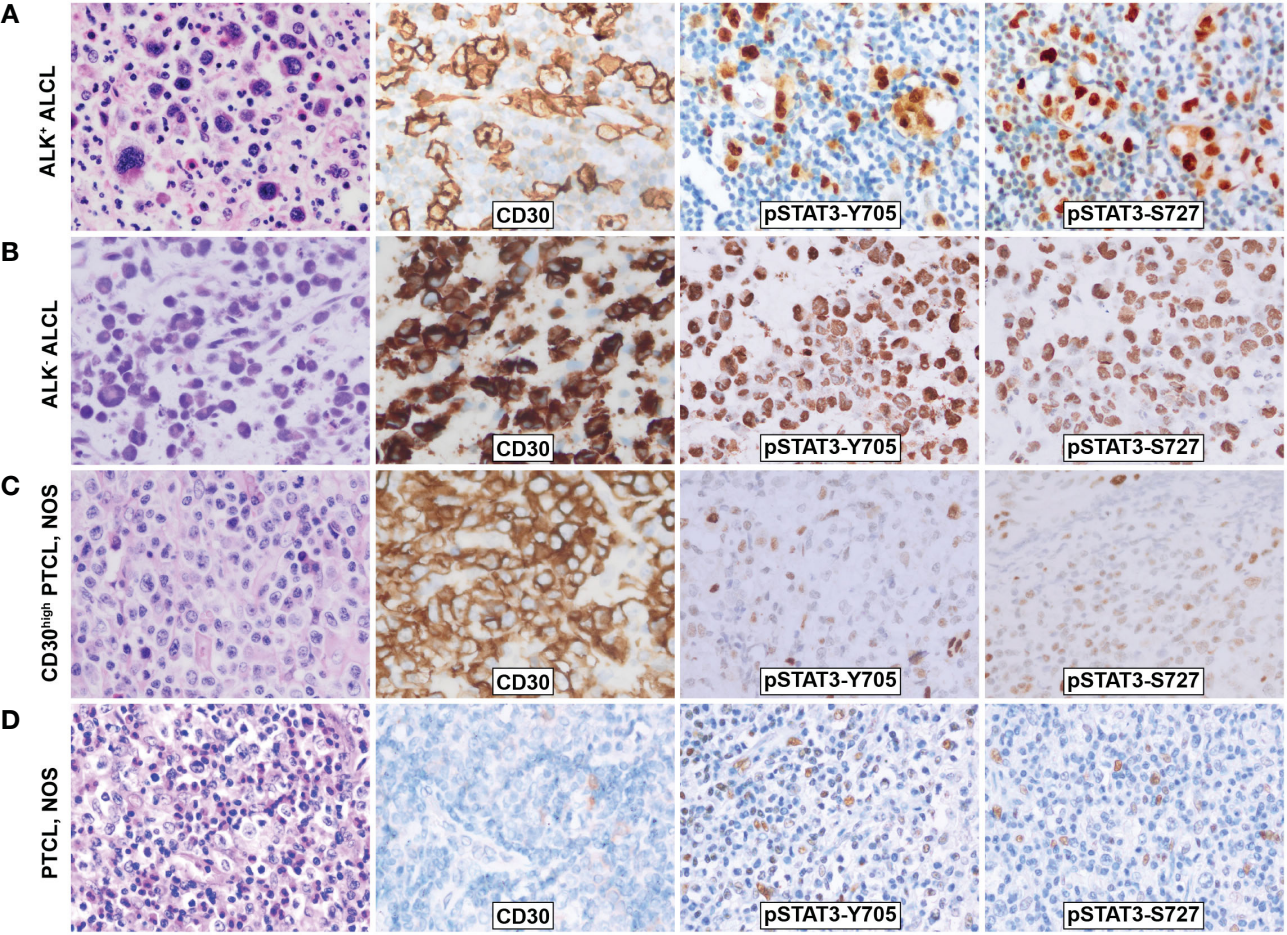
Morphology and phosphorylation of pSTAT3-Y705 and pSTAT3-S727 in ALCL and PTCL, NOS. **(A)**ALK^+^ ALCL; **(B)** ALK^-^ ALCL; **(C)** PTCL, NOS; **(D)** CD30^high^ PTCL, NOS. Original magnification: 400X.

Immunohistochemistry staining showed that both pSTAT3-Y705 and -S727 were highly expressed in the nuclei of tumor cells in both ALK^+^ and ALK^-^ ALCL, with diffuse and intense staining (**Figures 1A, B**). The majority of ALK^+^ ALCL cases (29/33, 88% for pSTAT3-Y705 and 30/33, 91% for pSTAT3-S727) and ALK^-^ ALCL cases (14/22, 64% for pSTAT-Y705 and 19/22, 86% for pSTAT3-S727) expressed pSTAT3-Y705 and pSTAT3-S727 in more than 70% of tumor cells. The median H-score of pSTAT3-Y705/S727 was 280/260 in ALK^+^ ALCL and 250/240 in ALK^-^ ALCL, respectively.

**Figure 2:**
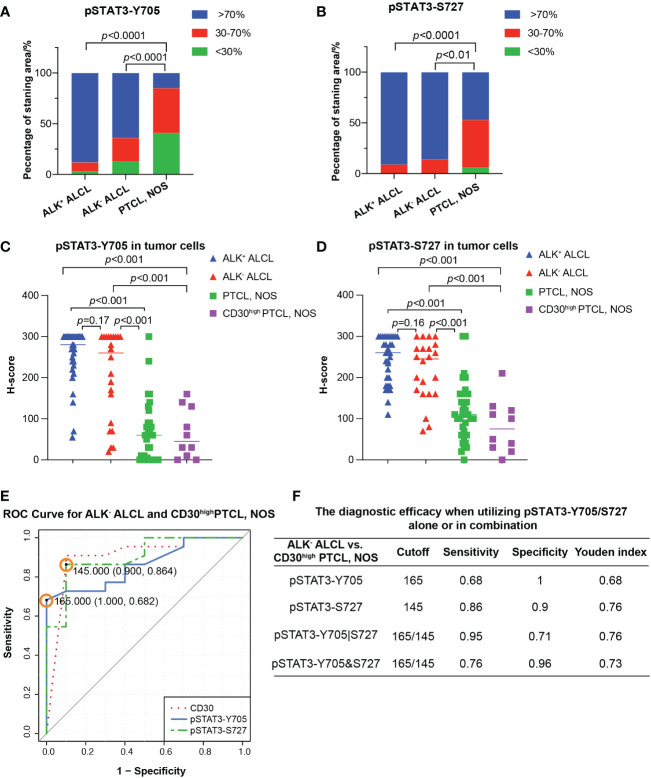
Differential diagnosis in ALCL vs. PTCL, NOS using pSAT3-Y705/S727. **(A, B)** Box plot demonstrating the proportion of staining area <30%, 30-70%, and High >70% for pSTAT3-Y705 **(A)** and pSTAT3-S727 **(B)** in ALK^+/-^ ALCL and PTCL, NOS. **(C, D)** Box plot for H-score of pSTAT3-Y705 **(C)** and pSTAT3-S727 **(D)** in tumor cells of ALK^+^/^-^ ALCL and PTCL, NOS, as well as in CD30^high^ PTCL, NOS. **(E)** Receiver operating characteristic (ROC) curves of using pSTAT3-Y705/S727 and CD30 to distinguish ALK^-^ ALCL and CD30^high^ PTCL, NOS; the point with the highest Youden index was marked in the ROC curve of pSTAT3-Y705/S727. **(F)** The diagnostic efficacy when utilizing pSTAT3-Y705/S727 alone or in combination. (Cutoff: the cutoff value was the H-score of pSTAT3-Y705/S727 which yielded the highest youden index (Sensitivity+Specificity-1); pSTAT3-Y705|S727: the parallel utilization of pSTAT3-Y705/S727, which indicates that if the neoplastic cells are positive for either pSTAT3-Y705 or pSTAT3-S727, the case is considered as positive; pSTAT3-Y705&S727: the serial utilization of pSTAT3-Y705/S727, which indicates that only when neoplastic cells are positive for both pSTAT3-Y705 and pSTAT3-S727, the case is considered as positive).

In contrast, PTCL, NOS cases had weak and limited expressin of pSTAT3-Y705 and pSTAT3-S727 in a subset of cells (**Figures 1C, D**). Only a proportion of PTCL, NOS cases expressed pSTAT3-Y705 (5/34, 15%) and pSTAT3-S727 (16/34, 47%) in more than 70% tumor cells (*p*<0.001 compared to ALK^+^ and ALK^-^ ALCL, **Figures 2A, B**). The median H-score of pSTAT3-Y705/S727 in PTCL, NOS was 60/100, significantly lower than that of ALK^+^ and ALK^-^ ALCL (*p*<0.001). Notably, CD30^high^ PTCL, NOS showed a similar pattern of pSTAT3-Y705/S727 positivity compared to other PTCL, NOS, with a median H-score of 45/75, significantly lower than that observed in ALK^+^ and ALK^-^ ALCL (*p*<0.001) (**Figures 2C, D**).

Next we investigated the potential use of pSTAT3-Y705 and pSTAT3-S727 in differentiating between ALK^-^ ALCL and CD30^high^ PTCL, NOS. Our results showed that all three markers had comparable diagnostic efficacy, with an AUC of 0.8614, 0.9023, and 0.9045 for pSTAT3-Y705, pSTAT3-S727, and CD30, respectively (pSTAT3-Y705 vs. CD30, *p*=0.67; pSTAT3-S727 vs. CD30, *p*=0.98; **Figure 2E**).To determine the optimal cutoff H-score values of pSTAT3-Y705 and pSTAT3-S727, we plotted ROC curves and calculated the Youden index. The cutoff H-score value for pSTAT3-Y705 was determined to be 165, while the cutoff H-score value for pSTAT3-S727 was determined to be 145 (**Figure 2E**). When the H-score value was above the cutoff value, a diagnosis of ALK^-^ ALCL was more likely than CD30^high^ PTCL, NOS.

Our analysis of the specificity and sensitivity of using pSTAT3-Y705 and pSTAT3-S727 alone or in combination for the differential diagnosis of ALK^-^ ALCL vs. CD30high PTCL, NOS is presented in **Figure 2F**. When used alone, pSTAT3-Y705 demonstrated better specificity, while pSTAT3-S727 demonstrated better sensitivity. When used in combination, parallel utilization of pSTAT3-Y705 and pSTAT3-S727 (Youden index, 0.76) could achieve equal diagnostic efficiency as pSTAT3-Y727 alone, with a higher sensitivity of 0.95, but a lower specificity of 0.71. Notably, pSTAT3-S727 alone demonstrated good diagnostic performance with balanced sensitivity and specificity (0.86 and 0.9, respectively, **Figure 2F**).

### The prognostic significance of pSTAT3-S727 as a biomarker

**Figure 3:**
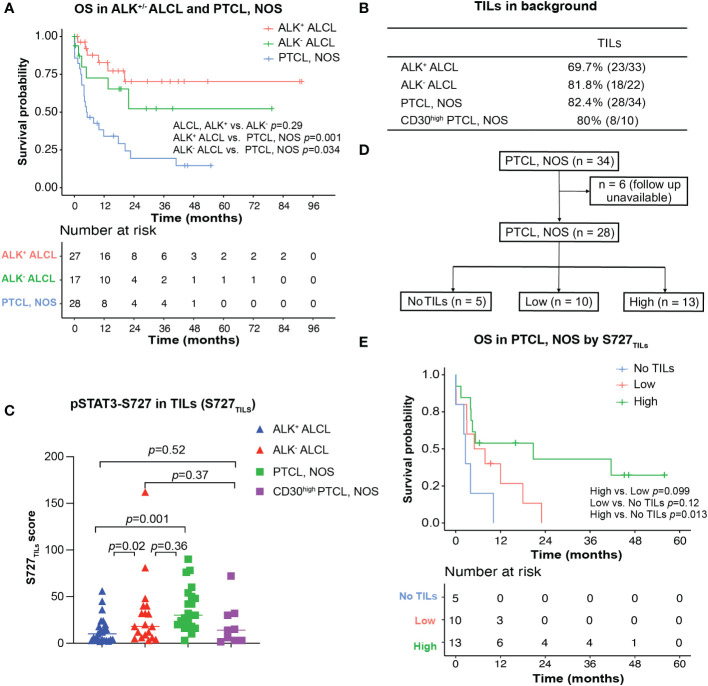
Survival analysis for ALK^+/-^ ALCL and PTCL, NOS patients. **(A)** Overall survival analysis of patients with ALK^+^/^-^ALCL and PTCL, NOS; **(B)** TILs could be observed in most ALK^+^ ALCL, ALK^-^ ALCL and PTCL, NOS cases; **(C)** Box plot for S727_TILs_ of ALK^+^/^-^ ALCL, PTCL, NOS, and CD30^high^ PTCL, NOS; **(D)** Flow chart illustrating the PTCL, NOS cases included in survival analysis in **(D)**; **(E)** Kaplan-Meier plot for PTCL, NOS patients with no TILs infiltration (“No TILs” group), with TILs expressing pSTAT3-S727 at a low level (“Low” group) and with TILs expressing pSTAT3-S727 at a high level (“High” group).

In our cohort, the median OS of both patients with ALK^+^ ALCL and patients with ALK^-^ ALCL was unreached. In contrast, patients with PTCL, NOS had a median OS of only 5.0 months (**Figure 3A**). We observed no significant difference in OS between patients with CD30^high^ and CD30^-/low^ PTCL, NOS (*p*=0.78, **Figure S1**).

**Figure 4:**
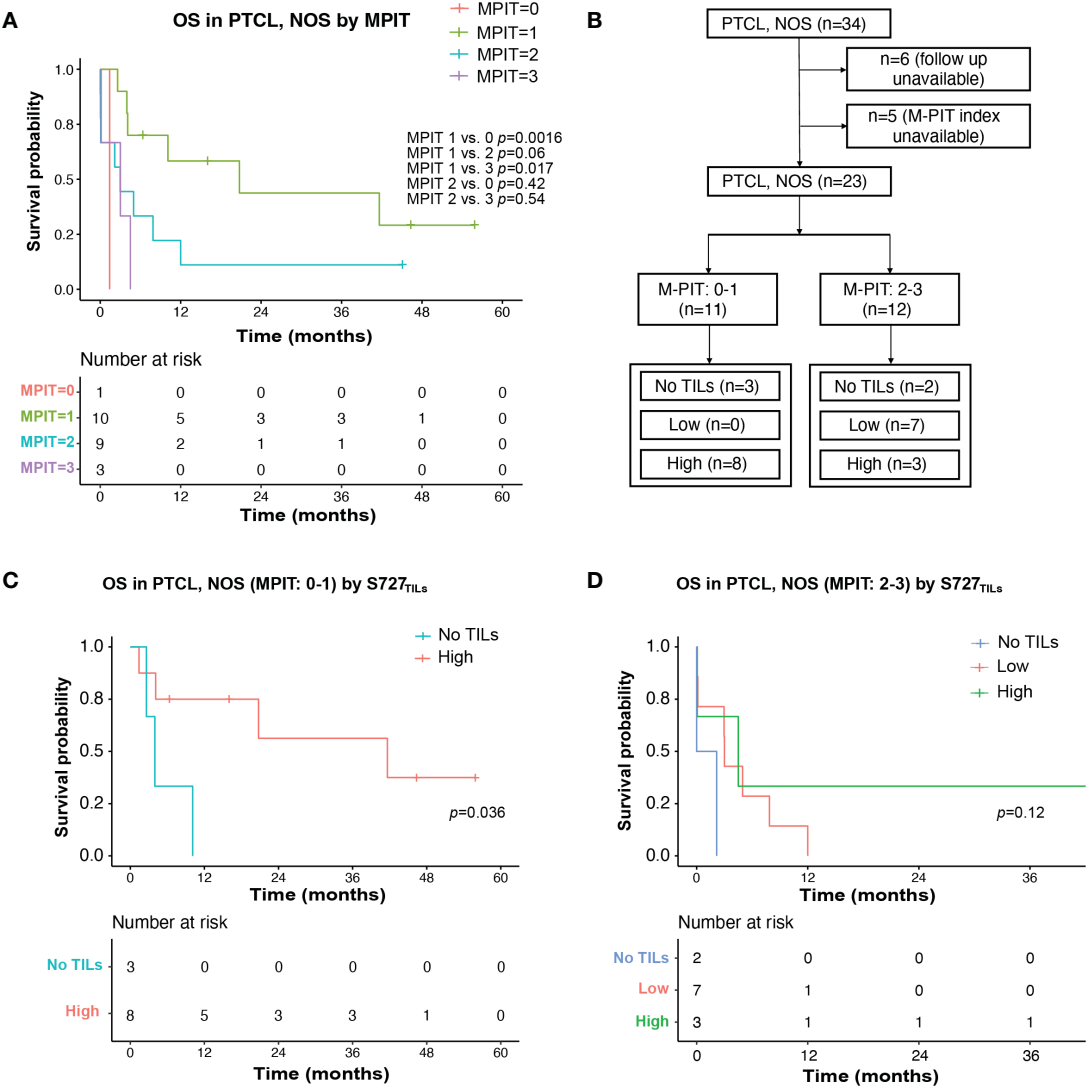
TILs expression level of pSTAT3-S727 (S727_TILs_) predicted PTCL, NOS patients’ prognosis independently of the M-PIT index. **(A)** Kaplan-Meier plot for PTCL, NOS patients with different M-PIT indexes; **(B)** Flow chart illustrating the PTCL, NOS cases included in survival analysis in **(C, D)**; **(C)** In PTCL, NOS patients of low risk (M-PIT: 0-1) patients with TILs expressing pSTAT3-S727 at a high level (“High”) had a better prognosis. **(D)** Kaplan-Meier plot for PTCL, NOS patients of high risk (M-PIT: 2-3) stratified by TILs appearance and their expression of pSTAT3-S727.

We then investigated whether any clinicopathological parameters could explain the poor prognosis of PTCL, NOS. Expression of CD30, pSTAT3-Y705, and pSTAT3-S727 in tumor cells, as well as various clinical and laboratory parameters, including bone marrow involvement, beta-2-microglobulin, platelets count, serum EBV DNA positivity, and decreased albumin, were not found to be predictive of prognosis in this study. However, the small sample size may have affected these results. We also analyzed whether known prognostic indices for PTCL, NOS, including the IPI, IPTCLP [International PTCL Project (21)], PIT [Prognostic Index for PTCL-U (22)], and MPIT [Modified prognostic index for PTCL, NOS (23)], could predict prognosis in our cohort. Only the PIT and M-PIT scores were found to be predictive (PIT, *p=*0.017, **Figure S2**; MPIT, *p*=0.042, **Figure 4A**).

Notably, while pSTAT3-Y705 was expressed primarily by lymphoma cells, we observed that pSTAT3-S727 was also present in background TILs, in addition to lymphoma cells (**Figures 1C, D**). As the infiltration of inflammatory cells in the background has been previously associated with poor prognosis in PTCL, NOS (24, 25), we evaluated the phosphorylation status of STAT3-S727 (S727_TILs_) and its significance in TILs (**Figures 3B, C**). Compared with ALK^+^ ALCL, the H score of S727_TILs_ was significantly higher in ALK^-^ ALCL (median: 18 vs. 10, *p*=0.02) and PTCL, NOS (median: 28.5 vs. 10, *p*=0.001). Additionally, the H score of S727_TILs_ was lower in CD30^high^ PTCL, NOS compared to CD30^-/low^ PTCL, NOS (median: 14.5 vs. 31; *p*=0.038; data not shown), and similar to that of both ALK^+^ (median: 14.5 vs. 10, *p*=0.52) and ALK^-^ ALCL (median: 14.5 vs. 19, p=0.37).

Next, we stratified PTCL, NOS patients into three subgroups based on the median H score of S727_TILs_ in PTCL, NOS (n=28, six patients were excluded due to loss of follow-up): those with no TILs (n=5), those with low S727_TILs_ (<28.5, n=10), and those with high S727_TILs_ (28.5, n=13) (**Figure 3D**). Patients with high S727_TILs_ H score had a significantly better OS compared to those with no TILs (**Figure 3E**); the 3-year OS rate was 43% vs. 0% (*p*=0.013), and there was a strong trend toward better OS than patients with low S727_TILs_ H score (*p*=0.099).

We investigated whether the prognostic impact of S727_TILs_ H score in PTCL, NOS patients was independent of the M-PIT index. To do this, we stratified patients into M-PIT low-risk group (0 or 1) and high-risk group (2 or 3) (**Figure 4B**). Among patients in the low-risk M-PIT group, those with high S727_TILs_ H score had a significantly better survival outcome than those with no TILs, with a median OS of 20.8 vs. 3.0 months and a 3-year OS of 39.8% vs. 0% (*p*=0.036, **Figure 4C**). However, the number of patients in the high-risk M-PIT group was too small for statistical analysis (**Figure 4D**).

### Flowcytometric analysis of pSTAT3-Y705/S727 in PTCL, NOS patients

**Figure 5:**
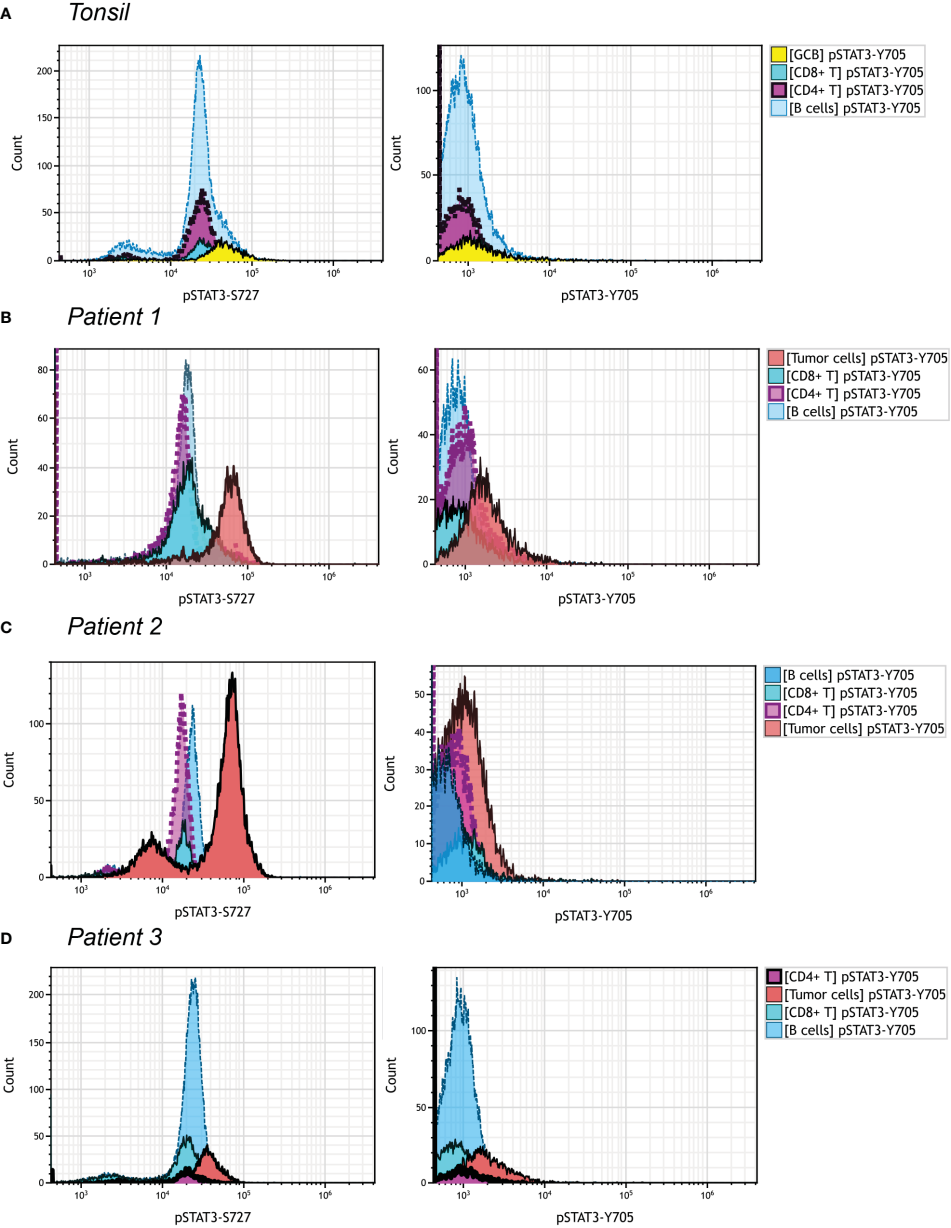
pSTAT3-Y705/S727 expression in PTCL, NOS by flowcytometric analysis. Different cell population was gated out as shown in **Figure S3**. The gate strategy of neoplastic cells in three PTCL, NOS patients was with reference to the initial immunophenotype at the time of diagnosis. **(A)** Histograms of pSTAT3-S727 (left) and pSTAT3-Y705 (right) expression of a tonsil sample; **(B–D)** Histograms of pSTAT3-S727 (left) and pSTAT3-Y705 (right) expression of *Patient 1* **(B)**, with neoplastic cell population being CD3^-^ and CD4^+^, *Patient 2* **(C)**, with that being CD3^bri^ and having enhanced FSC/SSC signals, and *Patient* **(D)**, with that being CD3^-^ and CD4^+^.

We managed to investigate the difference of expression pattern between pSTAT3-Y705 and pSTAT3-S727 in three PTCL, NOS patients, by multiparametric flowcytometric analysis. The gating strategy was demonstrated in **Figure S3** with reference to the immunophenotypes at the time of diagnosing. *Patient 1* and *Patient 3* were CD3^-^, CD4^+^ and *Patient 2* was CD3 bright with upregulated FSC/SSC signals. All three patients met the diagnosis criteria of PTCL, NOS without the expression of any Tfh marker. As shown in **Figure 5**, over half of B cells and nearly all CD4^+^ and CD8^+^ T cells in the tonsil sample were positive for pSTAT3-S727, with none of them positive for pSTAT3-Y705 (**Figure 5A**). The neoplastic cells of *Patient 1* and *Patient 2*, but not *Patient 3* had enhanced pSTAT-S727 signals and all three patients were negative for pSTAT3-Y705 expression by flowcytometric assay (**Figures 5B–D**). From the result of flowcytometric analysis, pSTAT3-S727 were constitutively positive in most T/B lymphocytes of normal tonsil and PTCL, NOS samples, and pSTAT3-Y705 were absent in all cell populations.

## Discussion

The diagnostic dilemma of ALK^-^ ALCL vs. CD30^high^ PTCL, NOS is one of the most challenging scenarios in the daily pathology practice. Although CD30 expression in PTCL, NOS is mostly weak and heterogeneous, some cases do express CD30 diffusely (80%) and strongly. Given the morphological and immunophenotypic overlap between ALK^-^ ALCL and PTCL, NOS, and no clinically available biomarker to differentiate them, the diagnosis between ALK^-^ ALCL and CD30^high^ PTCL, NOS could be challenging. We investigated whether phosphorylated STAT3 could help with the differential diagnosis and serve as a predictive biomarker by studying the expression of pSTAT3-Y705 and -S727 in 89 cases of ALK^+/-^ ALCL and PTCL, NOS, including 10 cases of CD30^high^ PTCL, NOS. Our results showed that pSTAT3-Y705 was more specific while pSTAT3-S727 was more sensitive in the differential diagnosis between ALK^-^ ALCL vs. CD30^high^ PTCL, NOS, with a relatively balanced sensitivity and specificity. Furthermore, the higher expression of pSTAT3-S727 in the TILs of PTCL, NOS was associated with a better prognosis.

Given the significance of STAT3 activation in tumorigenesis, researchers have undertaken different scoring strategies to evaluate pSTAT3 expression. However no cutoff value or scoring system has been widely accepted for the IHC staining of pSTAT3. In the present study, we used H-score, a well-established scoring system taking both staining extent and intensity into account, to assess pSTAT3-Y705 and -S727 expression. When studying the prognostic significance of pSTAT3-S727, considering the proportion of TILs in tumor areas might also affect patient prognosis, we scored S727_TILs_ by multiplying the H-score of S727_TILs_ by the percentage of TILs in tumor areas. However, a more concise and applicable semiquantitative scale may be needed in future clinical practice.

Despite differences in gene alterations between ALK^+^ ALCL and ALK^-^ ALCL, they share some deregulated transcription factors programs like STAT3 and NOTCH1 activation, IRF4, and c-MYC overexpression (9, 26–29). STAT3 activation has been reported in ALK^+^ ALCL and ALK^-^ ALCL as a well-defined oncogenic driver through different machanisms (9, 12–14, 29–31). In the present study, we found that both pSTAT3-Y705 and -S727 were diffusely expressed by tumor cells in ALCL, irrespective of ALK status; though pSTAT3-Y705 was more specific and pSTAT3-S727 is more sensitive. However, they were less frequently and variably expressed in PTCL, NOS, implying they could be reliable differential biomarkers between ALCL vs. PTCL, NOS. When narrowing down to differential diagnosis between ALK^-^ ALCL and CD30^high^ PTCL, NOS, considering a minority (13%) of ALK^-^ ALCL expressing pSTAT3-Y705 in a very low level (<30%), pSTAT3-S727 appears to be a better choice, with a sensitivity of 0.86 and specificity of 0.9. Our results suggest that pSTAT3-S727 IHC staining could help distinguish ALK^-^ ALCL from PTCL, NOS, even when CD30 is diffusely positive: if the H-score of pSTAT3-S727 expression surpasses 145, a diagnosis of ALK^-^ ALCL is favorable. This IHC-based classifier guides the differential diagnosis between ALK^-^ ALCL and CD30^high^ PTCL, NOS with a sensitivity of 0.86 and specificity of 0.9, suggesting pSTAT3-S727 is a promising differential biomarker for ALK^-^ ALCL vs. CD30^high^ PTCL, NOS.

For what reason the STAT3 phosphorylation status in PTCL, NOS differs from ALK^-^ ALCL remains unclear. In ALK^+^ ALCL, ALK kinase constitutively drives STAT3 phosphorylation (30). However, in ALK^-^ ALCL, only ~20% of ALK- ALCLs have activate mutations of JAK1 and/or STAT3 genes (9), which could not explain the high rate of pSTAT3 positivity both in our study and other researchers’ work (17, 18, 32). Additionally, compared with ALK^+/-^ ALCL, a lower proportion of PTCL, NOS expresses pSTAT3, reported also by other researchers (14, 18). In line with this, the mutations JAK1 and/or STAT3 was a rare event in PTCL, NOS: M. Rodríguez et al. detected JAK1/STAT3 mutations in 0/14 PTCL, NOS patients (33). However, E. Andersson et al. reported a higher rate of STAT3 mutations in PTCL, NOS in comparison with ALK^-^ ALCL (17% vs. 13%), and a lower rate of JAK1/3 mutations (0 vs. 15%) (14). In our study, five PTCL, NOS and one ALK^-^ ALCL cases were subjected to DNA sequencing. All patients, including those who were positive for pSTAT3-Y705 and/or pSTAT3-S727 were negative for JAK1/STAT3 mutations. The low prevalence of JAK1/STAT3 mutations makes the positivity rate vary in different studies. As of now, there is still a lack of positive association between pSTAT3 expression and JAK1/STAT3 mutations. Other signaling pathway was suggested to contribute to phosphorylation of STAT3. For example, U. Rozovski et al. reported that in chronic lymphocytic leukemia (CLL), the constitutive phosphorylation of STAT3 was induced by CK2-BLNK-CD5 complex (34). Further studies are needed to explore the mechanisms of STAT3 phosphorylation other than JAK1/STAT3 mutations.

Our results showed that pSTAT3-Y705 and -S727 in tumor cells were not prognostically predictive in ALCL patients. For PTCL, NOS patients, only the expression of S727_TILs_ was associated prognosis: those with higher S727_TILs_ H score tended to have a higher 3-year OS rate than those with a lower S727_TILs_ H score (43% vs. 0%, *p*=0.013). This result is plausible, given that PTCL, NOS is characterized by abundant background inflammatory cells including TILs. The interplay between tumor cells and the background immune cells mediated by cytokines release or exosome intake might contribute to the poor prognosis of PTCL, NOS patients. Our results suggested that the activation of STAT3 might contribute to the prognostic significance of TILs in PTCL, NOS patients. Furthermore, we found that high S727_TILs_ H score was enriched in PTCL, NOS patients with low MPIT risk factors compared to the low S727_TILs_ group (data not shown, *p*<0.05). MPIT model had a good performance in predicting patient prognosis in our study. This suggests that S727_TILs_ could help stratify PTCL, NOS patients with low MPIT index. However, the small sample size of the study warrants cautious interpretation of the difference in OS between the subgroups based on S727_TILs_ H score. Larger studies are needed to confirm findings and provide more robust evidence.

The discrepancy of pSTAT3-Y705 and pSTAT3-S727 was observed in our study, with pSTAT3-Y705 expressed mainly by lymphoma cells and pSTAT3-S727 also positive in background besides lymphoma cells. This was verified in our flowcytometric analysis, where pSTAT3-S727 were constitutively positive in most T/B lymphocytes of normal tonsil and PTCL, NOS samples, and pSTAT3-Y705 were absent in all cell populations. There was still a lack of knowledge about this phenomena. As we know, the canonical IL-6/JAK signaling pathway activates STAT3 at Y705 and S727 simultaneously, and S727 phosphorylation is indispensable for STAT3 activation to its fullest extent (35). However, unlike Y705, S727 was activated also by alternative upstream signaling pathways. For example, the TLR-4 recruited TBK-1 to phosphorylate STAT3 S727 in macrophage cells (36). Additionally, S727 was phosphorylated by MAPK in embryonic stem cells (37), correlated with YAP1 in glioma (38) and PIK3K-AKT-mTOR in colorectal cancer cells (38). In summary, unlike Y705, S727 was more frequently activated by alternative upstream signaling instead of IL-6/JAK signaling, and represented an non-canonical way of STAT3 activation. Furthermore, pSTAT3-Y705 and pSTAT3-S727 might play different roles in various conditions. Liang et al. reported that only STAT3 Y705 but not S727 promoted cancer cell EMT and metastasis through slug (39). Mandal et al. reported that overexpression of CK2 reduced Stat3 S727 phosphorylation enhanced tumorigenicity, but conversely phosphorylated Stat3 Y705 in similar conditions (40). The uncoupling of Y705 and S727 was observed in Kaposi sarcoma, in which case, the viral infection exclusively induced S727 phosphorylation to foster a unique chronic inflammatory environment by activating mitogen-activated kinase-activated protein (MAPKAP) kinase 2 (MK2) chronic inflammatory (41). We still have no evidence to explain the discrepancy of Y705 and S727 in our case, however we suggested the unique tumor microenvironment consisting of unknown released cytokines and microorganisms infection like Epstein-Barr virus (EBV), could be the reasons.

In summary, we found that ALCL and PTCL, NOS show different activation status of STAT3. Based on this difference, we established an IHC classifier to help differential diagnosis between ALK^-^ ALCL and PTCL, NOS with diffuse CD30 expression. Furthermore, we found that a higher expression of pSTAT-S727 by TILs in PTCL, NOS predicts a better prognosis.

## Data availability statement

The original contributions presented in the study are included in the article/**Supplementary Material**. Further inquiries can be directed to the corresponding author.

## Ethics statement

The studies involving human participants were reviewed and approved by the ethics committee of Affiliated Hospital of Xuzhou Medical University. Written informed consent for participation was not required for this study in accordance with the national legislation and the institutional requirements.

## Author contributions

HL designed the study. CX and WW designed the study, performed the experiments, analyzed the data and drafted this manuscript. ZW, XF, CL, and JL reviewed part of the slides. LX, GL, and HS performed the IHC assays. YG, NL, DL, MF, and YW collected the clinical information of the patients. SH helped to improve the manuscript. All authors contributed to the article and approved the submitted version.

## Supplementary material

Supplementary Figure 1 Univariate survival analysis for PTCL, NOS patients with different clinical or pathological risk factors.

Supplementary Figure 2 Kaplan-Meier plot for PTCL, NOS patients according to Ann Arbor staging system, International Prognostic Index (IPI), Prognostic Index for PTCL, NOS (PIT), and International PTCL Project (IPTCLP).

Supplementary Figure 3 Gating strategy of the tonsil and three PTCL, NOS samples in .
