# Supplementary material for: Phosphorylated STAT3 as a potential diagnostic and predictive biomarker in ALK- ALCL vs. CD30high PTCL, NOS
Source: Front Immunol. 2023 Jun 14;14:1132834. doi: 10.3389/fimmu.2023.1132834 (PMC10303105; doi:10.3389/fimmu.2023.1132834)
Supplement: Supplementary file 1 [file Table_1.docx]

| **Patient No.** | **Diagnosis** | **CD30** | **pSTAT3-Y705** | **pSTAT3-S727** | **NGS Results** |
| --- | --- | --- | --- | --- | --- |
| 1 | PTCL, NOS | 45 | 240 | 140 | No aberrations detected |
| 2 | PTCL, NOS | 0 | 0 | 90 | ARID1B c.4562G>A(R1521H)  EP300 c.4142A>G(p.Y1381C) |
| 3 | PTCL, NOS | 0 | 300 | 300 | DNMT3A c.2645G>A(p.R882H) |
| 4 | PTCL, NOS | 210 | 140 | 30 | NOTCH1 c.7326_7327insTGAAGC  (p.D2442_V2443ins*)  CHD8 c.5353G>A(p.E1785K)  CTCF c.1122_1134delinsCC(p.G375Lfs*30)  DNM2 c.188T>A(p.I63N), c.1153C>T(p.R385*)  EZH2 c.2018A>T(p.N673I)  KDM6A c.3332_3333insAGTGTTCTCC  (p.V1113Ffs*41)  PHF6 c.821G>A(p.R274Q)  SPEN c.7156C>T(p.Q2386*)  SUZ12 c.301C>T(p.R101*)  ZAP70 c.959G>T(p.S320I) |
| 5 | PTCL, NOS | 300 | 300 | 260 | PTPN2 c.53G>A(p.W18*) |
| 6 | ALK- ALCL | 260 | 30 | 40 | CD58 c.454C>T(p.R152*)  TP53 c.916C>T(p.R306*)  ALK c.1276A>G(P.S426G)  MAP3K1~TBL1XR1 MAP3K1:exon1~TBL1XR1:exon6 |

**Table S1: NGS results of PTCL, NOS and ALK^-^ ALCL**
