# Supplementary figures and images for: Phosphorylated STAT3 as a potential diagnostic and predictive biomarker in ALK- ALCL vs. CD30high PTCL, NOS
Source: Front Immunol. 2023 Jun 14;14:1132834. doi: 10.3389/fimmu.2023.1132834 (PMC10303105; doi:10.3389/fimmu.2023.1132834)

**Table S2: DNA sequencing panel of 103 genes related with T/NK cell lymphoma**


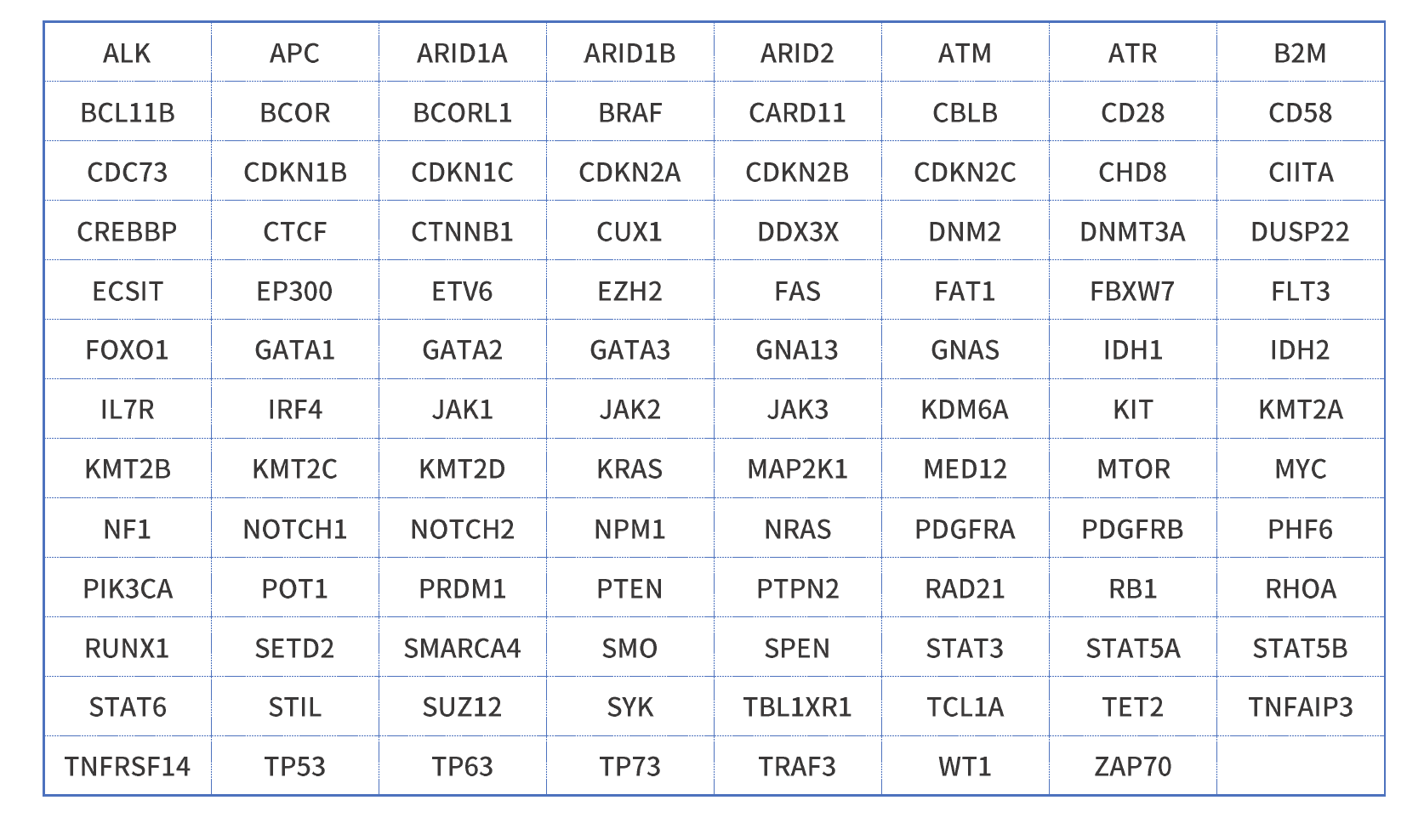

Supplement: Supplementary file 2 [file Table_2.docx]
