# Supplementary material for: Phosphorylated STAT3 as a potential diagnostic and predictive biomarker in ALK- ALCL vs. CD30high PTCL, NOS
Source: Front Immunol. 2023 Jun 14;14:1132834. doi: 10.3389/fimmu.2023.1132834 (PMC10303105; doi:10.3389/fimmu.2023.1132834)

Tonsil+Cocktail+pSTAT3
New Protocol 4 - Plot Sheet 1

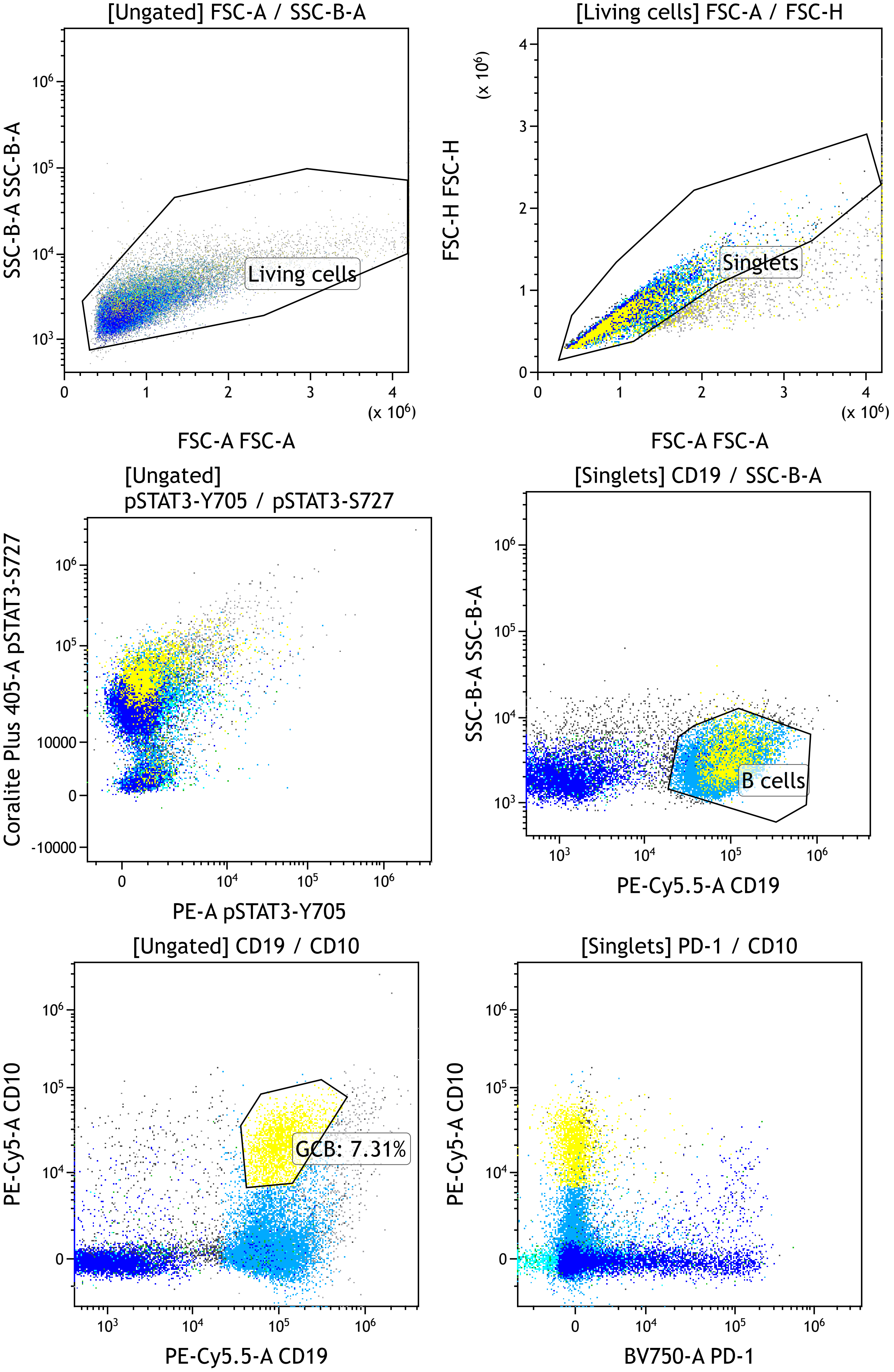

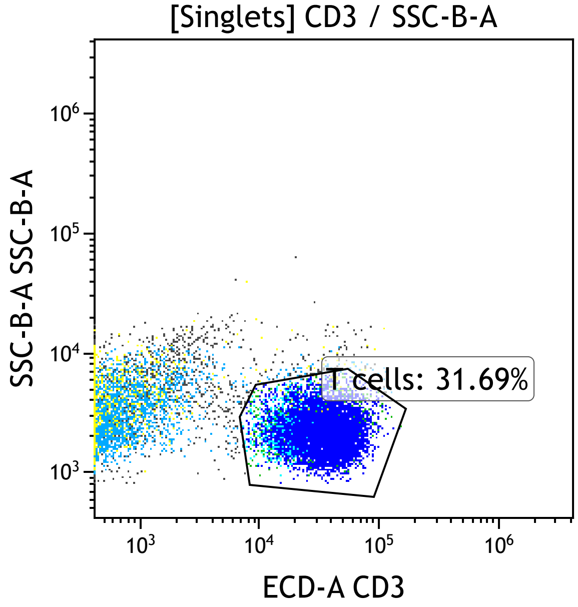
[Singlets] CD3 / SSC-B-A
SSC-B-A SSC-B-A
ECD-A CD3
T cells: 31.69%
10^3
10^4
10^5
10^6

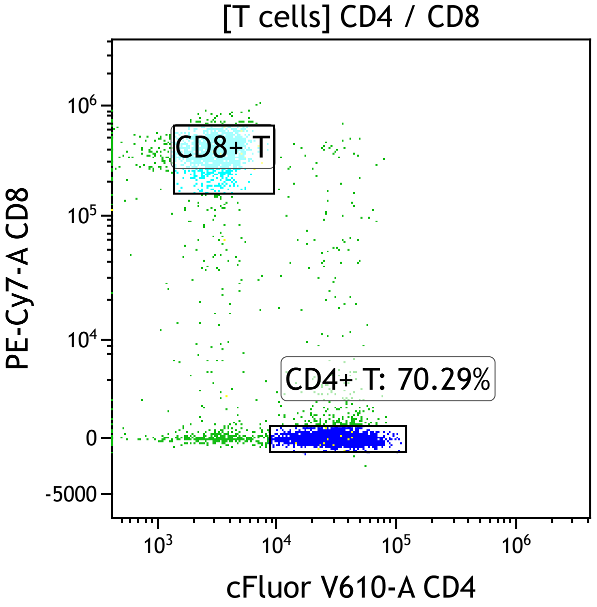
[T cells] CD4 / CD8
PE-Cy7-A CD8
cFluor V610-A CD4
CD8+ T
CD4+ T: 70.29%
10^6
10^5
10^4
0
-5000
10^3

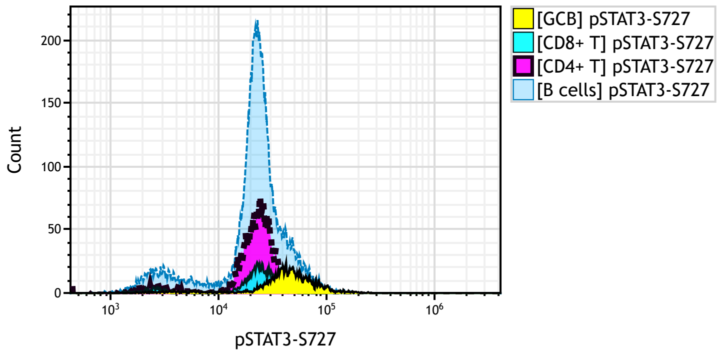
Count
pSTAT3-S727
200
150
100
50
0
[GCB] pSTAT3-S727
[CD8+ T] pSTAT3-S727
[CD4+ T] pSTAT3-S727
[B cells] pSTAT3-S727

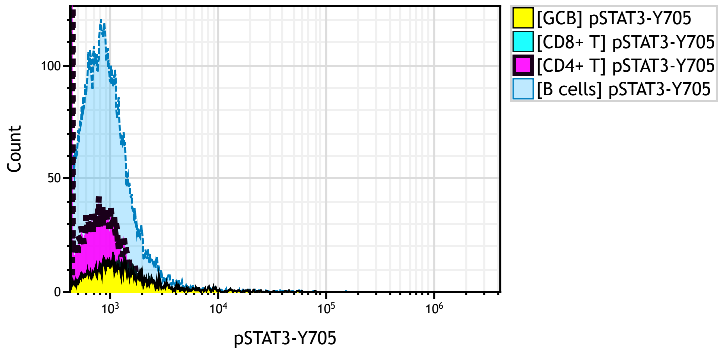
Count
pSTAT3-Y705
100
50
0
[GCB] pSTAT3-Y705
[CD8+ T] pSTAT3-Y705
[CD4+ T] pSTAT3-Y705
[B cells] pSTAT3-Y705

Patient1+Cocktail+pSTAT3
New Protocol 4 - Plot Sheet 1

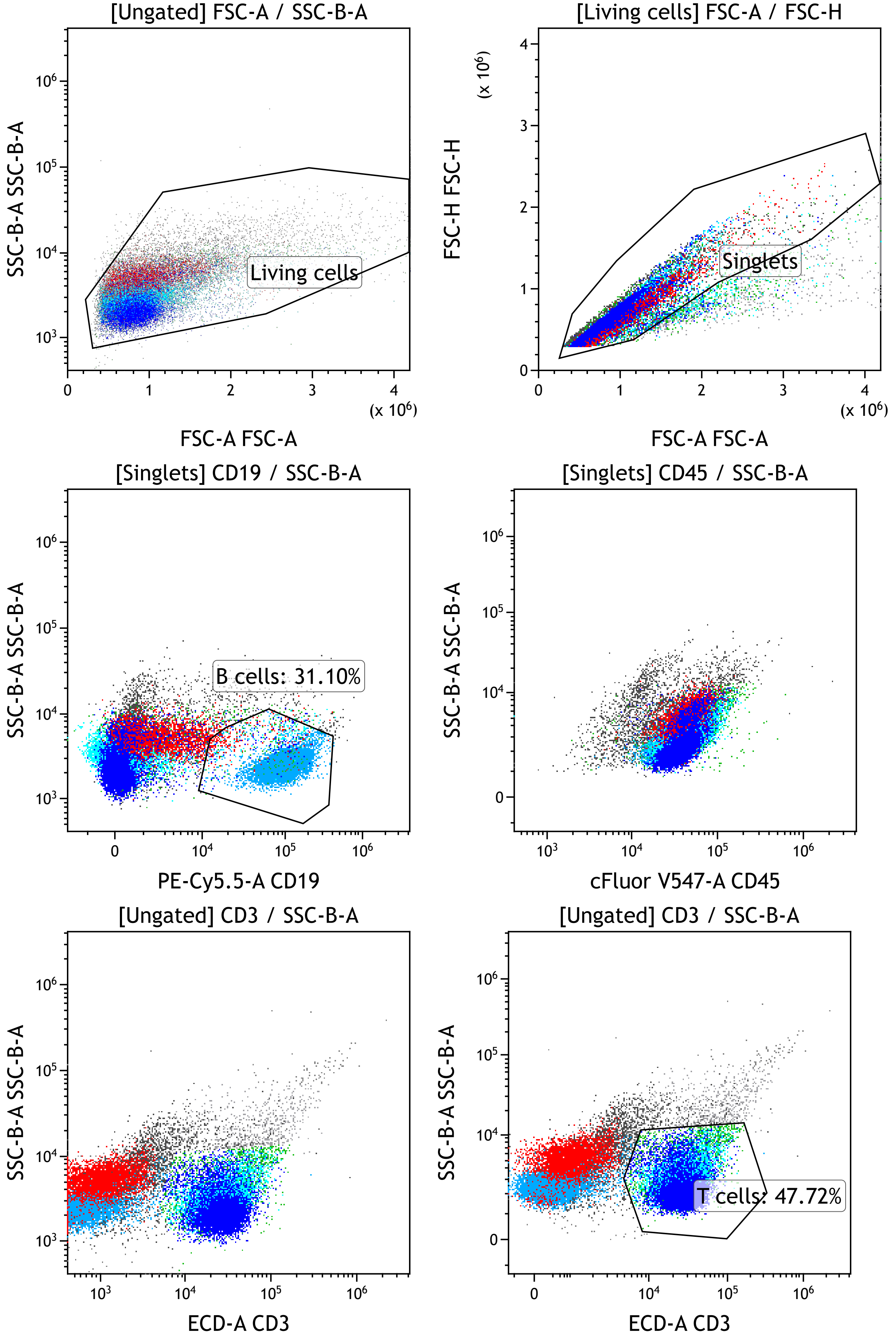

Patient1+Cocktail+pSTAT3
New Protocol 4 - Plot Sheet 1

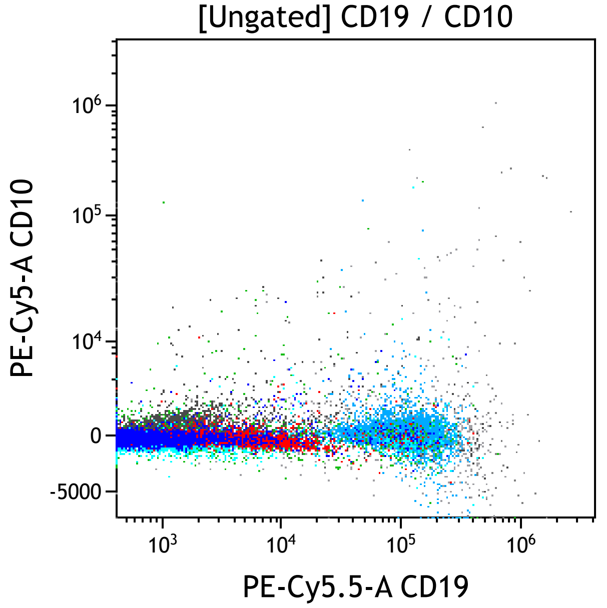

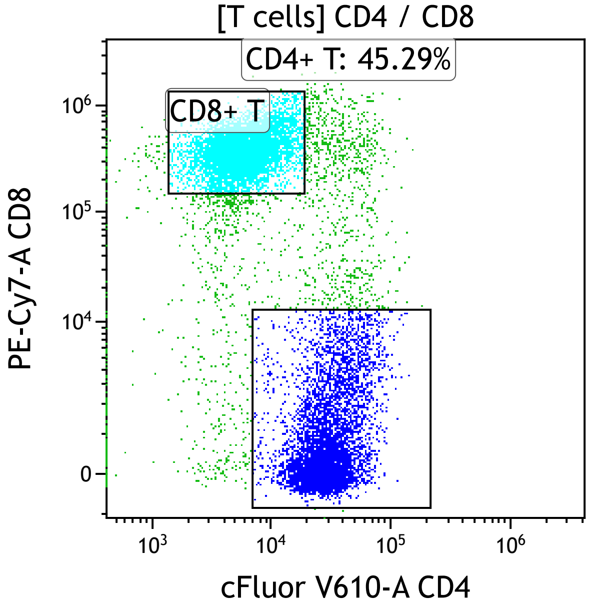

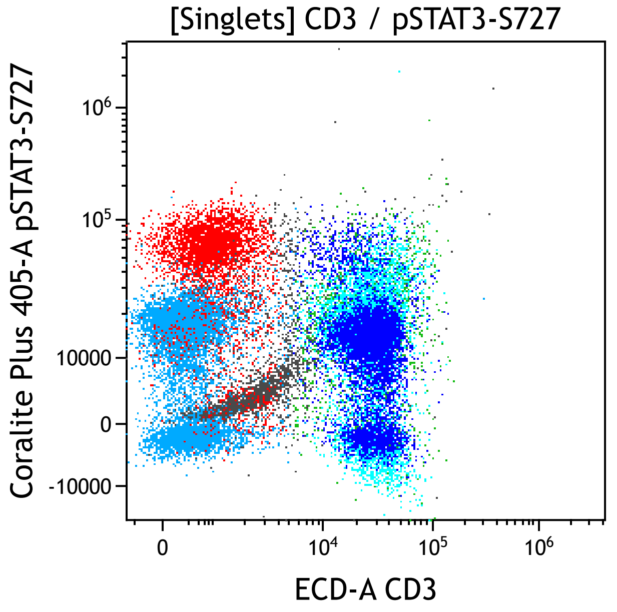

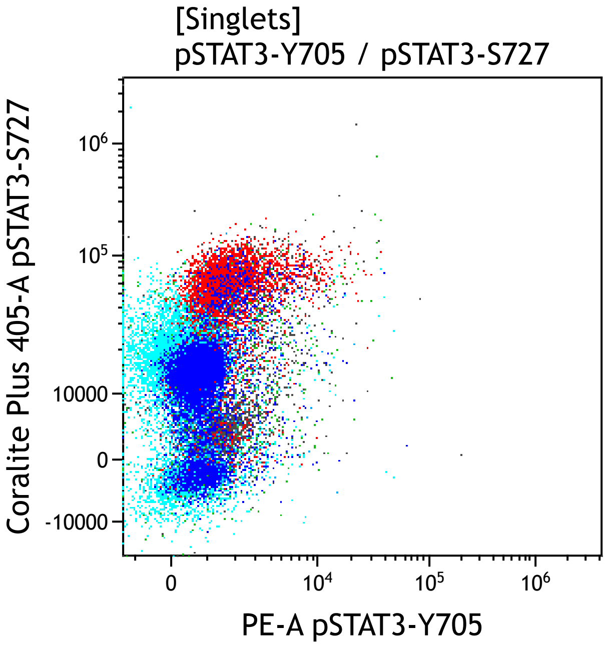

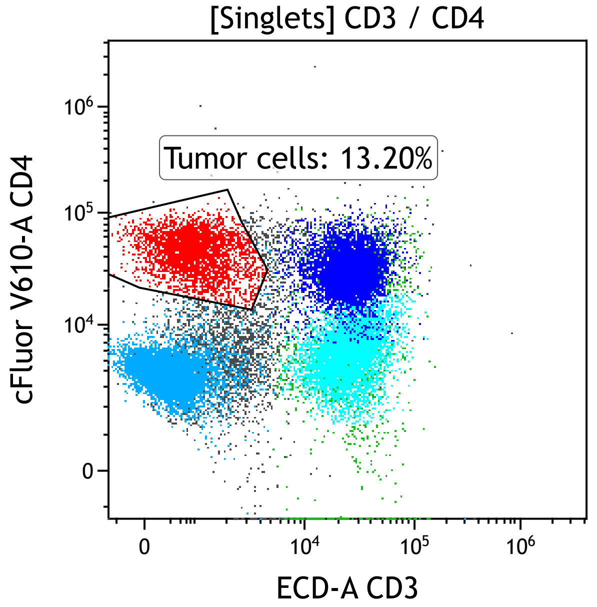

Patient1+Cocktail+pSTAT3
New Protocol 4 - Plot Sheet 1

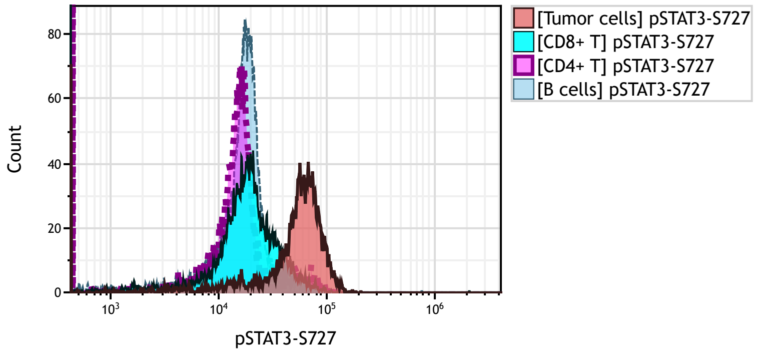

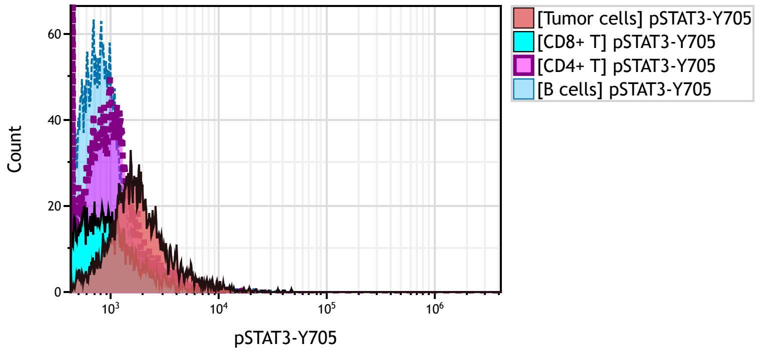

Patient2+Cocktail+pSTAT3

New Protocol 4 - Plot Sheet 1

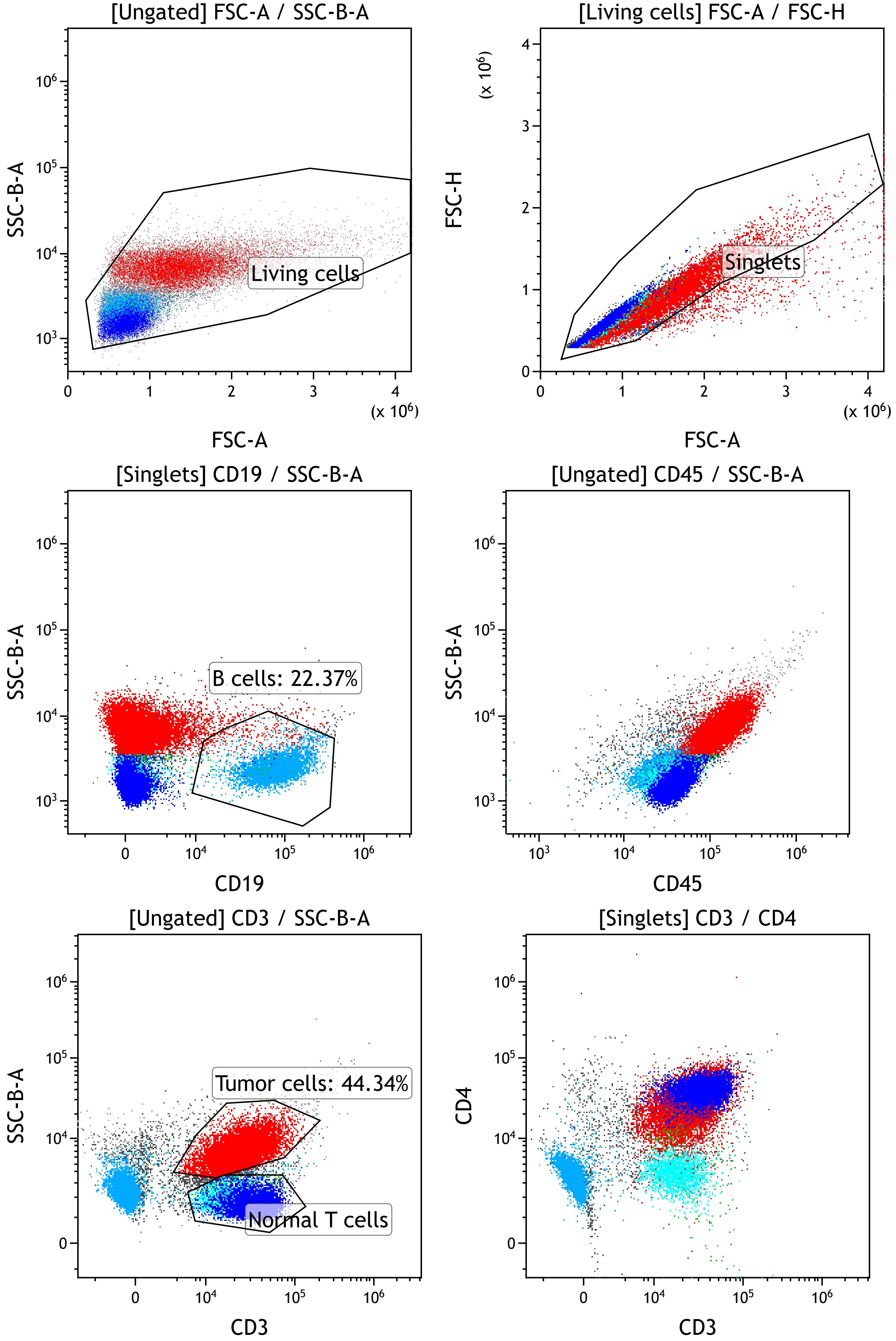

Patient2+Cocktail+pSTAT3
New Protocol 4 - Plot Sheet 1

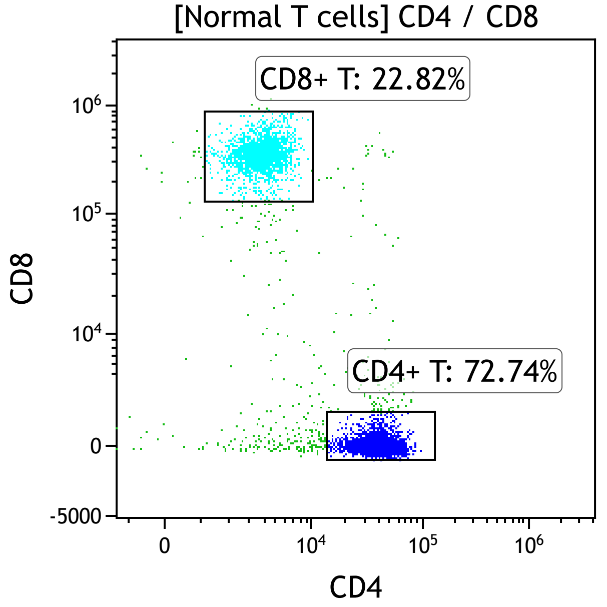

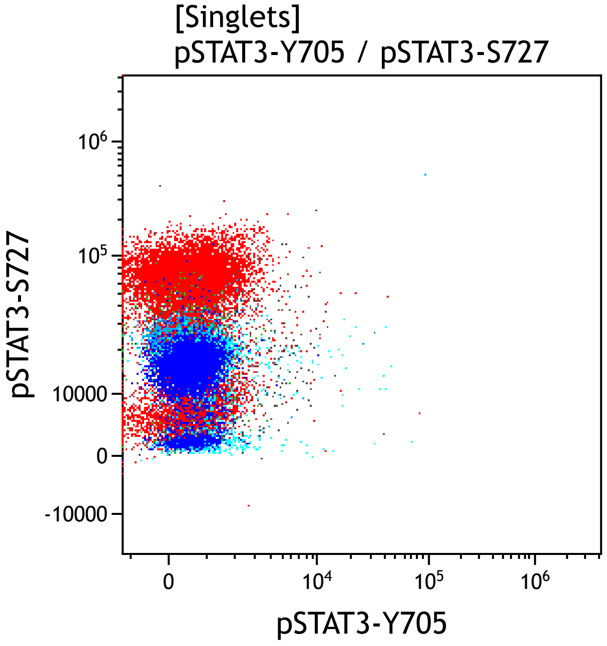

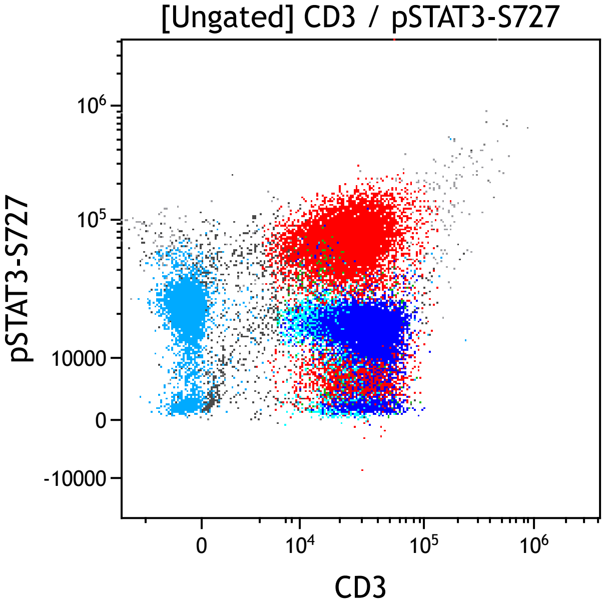

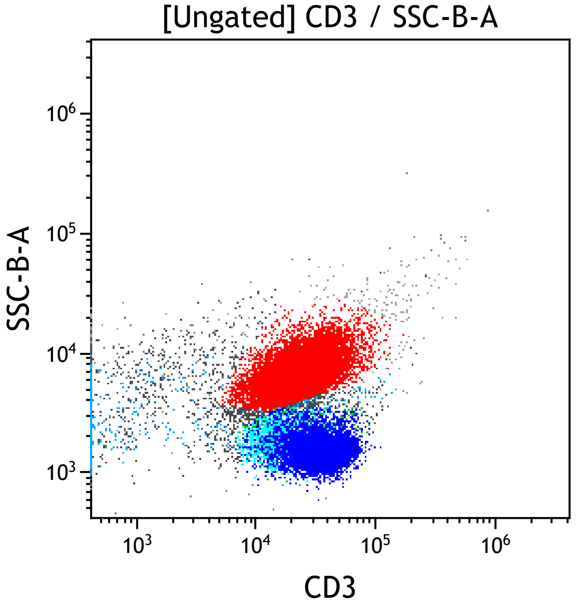

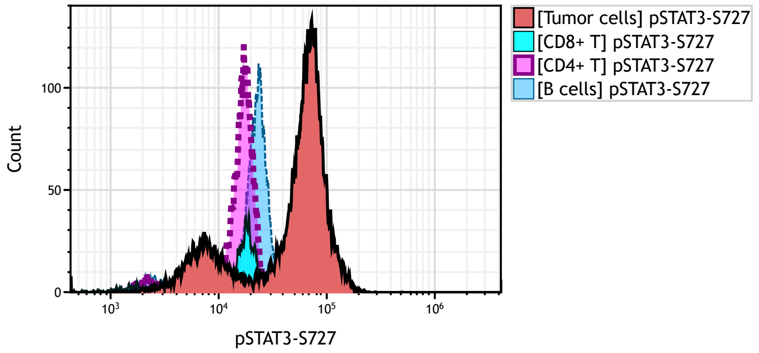

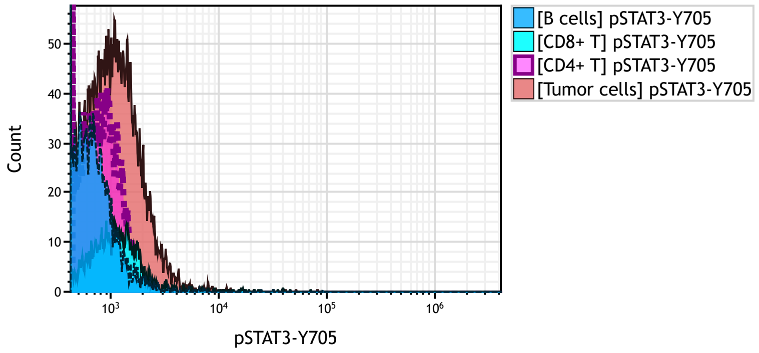

Patient3+Cocktail+pSTAT3
New Protocol 4 - Plot Sheet 1

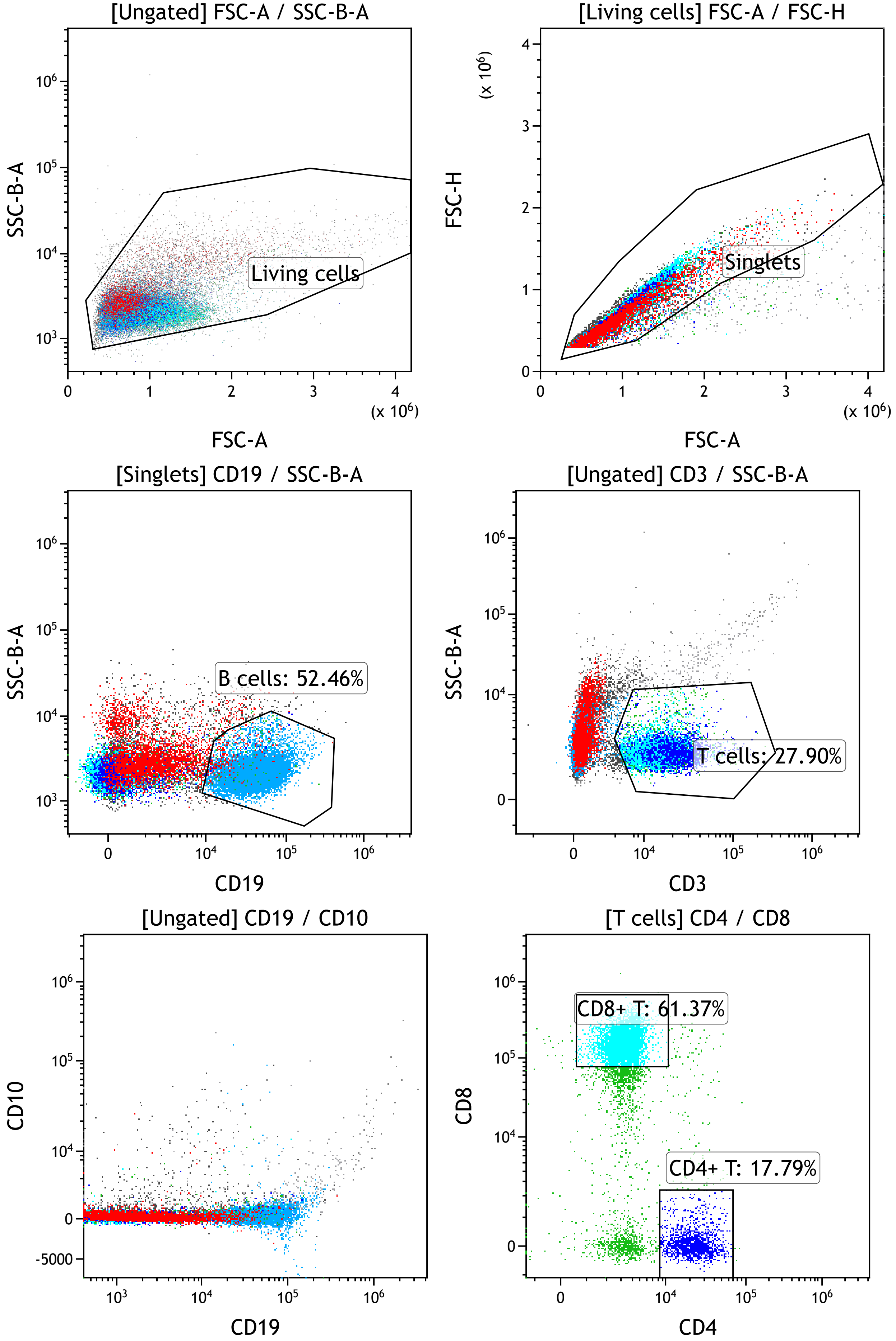

Patient3+Cocktail+pSTAT3
New Protocol 4 - Plot Sheet 1

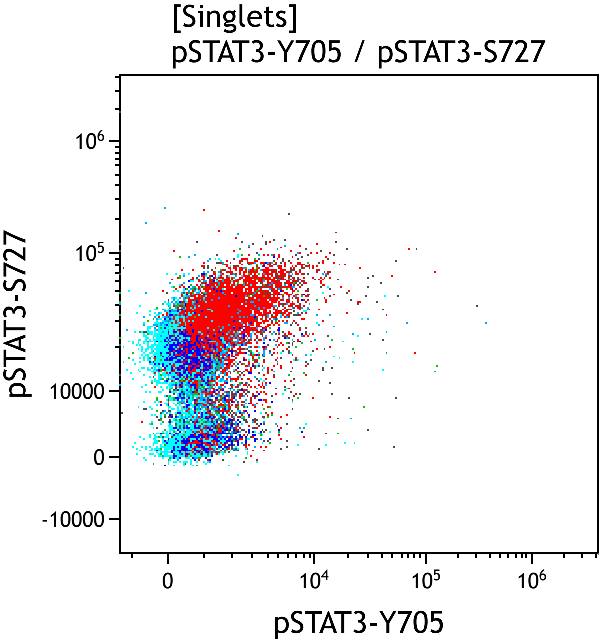

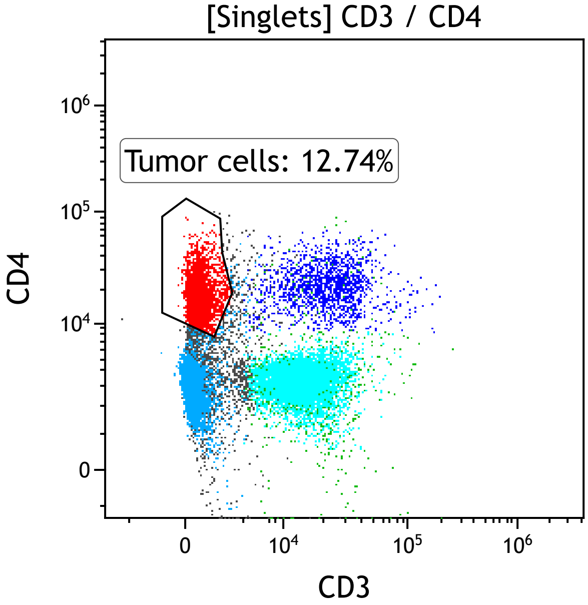

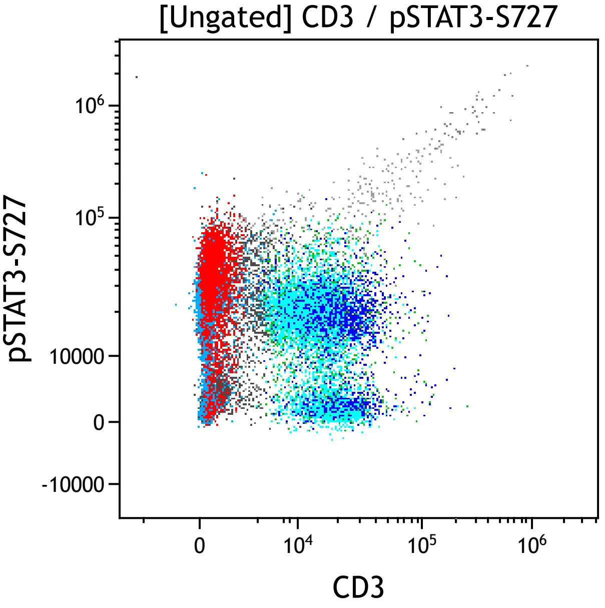

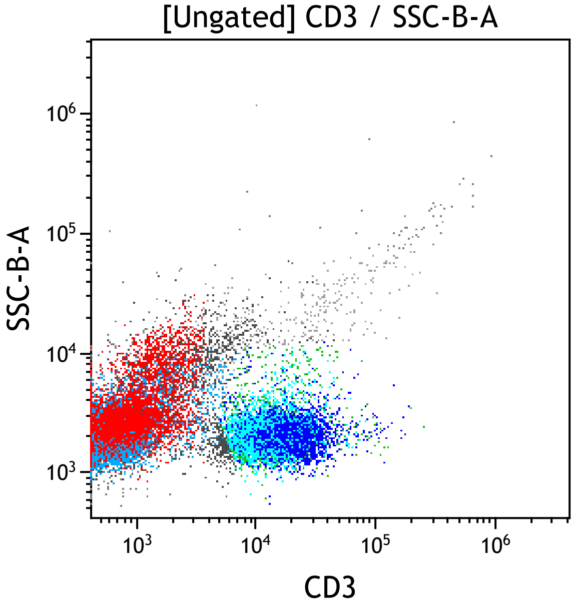

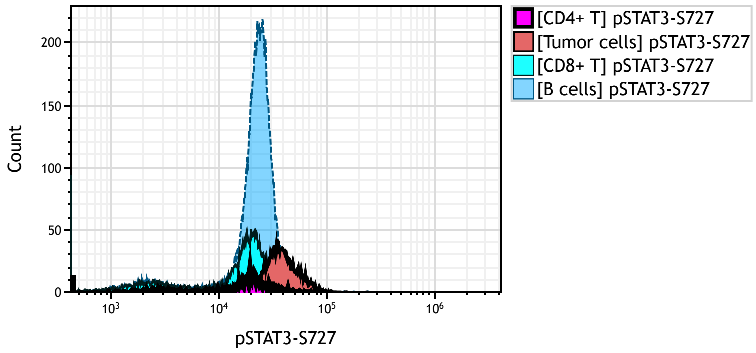

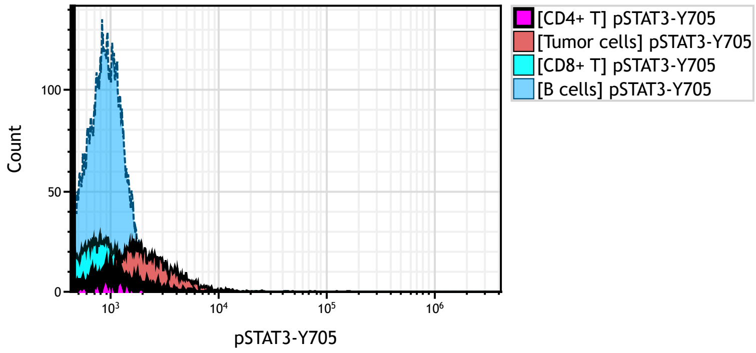

Supplement: Supplementary Figure 1 — Univariate survival analysis for PTCL, NOS patients with different clinical or pathological risk factors. [file DataSheet_1.pdf]
